# Application of a YOLOv8-Based Monocular Ground-Plane Intersection Method to Pothole Detection

**DOI:** 10.3390/s26154701

**Published:** 2026-07-23

**Authors:** Yuanbo Zhu, Qing He, Haoran Lin, Xiwang Cui

**Affiliations:** School of Instrumentation Science and Opto-Electronics Engineering, Beijing Information Science and Technology University, Shahe Campus, Beijing 100192, China; zyb_0609@163.com (Y.Z.); 17340798095@163.com (H.L.); cuixiwang@bistu.edu.cn (X.C.)

**Keywords:** monocular distance estimation, road potholes, ground plane intersection method, intelligent vehicles

## Abstract

Targeting the problem of road pothole detection and distance estimation under controlled experimental conditions, this paper proposes a method for pothole identification and ranging based on monocular vision and the ground-plane intersection method. The camera is calibrated using Zhang Zhengyou’s checkerboard calibration method to obtain its intrinsic and extrinsic parameters along with distortion coefficients. This is combined with the YOLOv8 object detection model to achieve pothole identification. Furthermore, a pothole distance estimation model based on the ground-plane intersection method is constructed. The model is validated through sandbox experiments conducted on a smart-car platform equipped with a Raspberry Pi 5B. Experimental results demonstrate the preliminary feasibility of the proposed method for short-range pothole detection and distance estimation in a controlled sandbox environment, while further validation under more complex real-road conditions is still needed.

## 1. Introduction

Smart cars need to detect potholes on the road in a timely manner and take actions such as slowing down, detouring, or avoiding them. This can significantly reduce accident risks while improving travel efficiency [1,2,3]. Therefore, combining pothole recognition with intelligent driving technology has become an important direction for ensuring road safety and operational efficiency. The variable shapes of potholes make them difficult for traditional sensors to identify and detect accurately. In this context, the emergence of intelligent driving technology offers new approaches to solve this problem.

Traditional smart cars mostly rely on conventional detection methods like infrared and ultrasonic sensors to achieve obstacle avoidance [4,5,6,7]. While these sensors perform well in detecting large obstacles such as pedestrians and vehicles with good accuracy and response speed, they cannot effectively detect ground potholes. Meanwhile, accelerometers only sense vibrations when the car passes over a pothole, failing to provide early warning. Although high in precision, sensors like millimeter-wave radar and LiDAR have weak reflections from small-scale, low-lying, non-metallic concave targets like potholes, making accurate shape identification difficult [8,9].

Compared to traditional methods, computer vision provides a new direction for further research [10,11]. By equipping cameras and object detection algorithms, smart cars can perceive their surroundings in real time, identify potholes and other targets, and take appropriate actions. However, road pothole detection still faces challenges caused by variations in road texture, illumination, and target appearance. These factors increase the difficulty of accurately identifying potholes and estimating their distance. Therefore, developing a reliable pothole detection and ranging method under controlled experimental conditions can provide a useful basis for further validation in more complex road scenarios.

Object detection is a key task in computer vision. Its goal is to identify and locate multiple objects within an image. In recent years, with the development of deep learning algorithms, object detection methods based on Convolutional Neural Networks (CNNs) have made significant progress. Current object detection algorithms are mainly divided into two categories. One category includes two-stage detection algorithms represented by Fast R-CNN [12] and Faster R-CNN [13]. The other category is one-stage detection algorithms, represented by YOLO. These algorithms predict an object’s location and class directly from the input image, skipping the complex region proposal step. This gives them a clear advantage in detection speed [14]. Because one-stage algorithms do not rely on pre-defined anchors, they perform well in detecting small objects. For resource-constrained smart cars, using a one-stage algorithm balances accuracy and speed. This helps ensure the safe travel of the smart car along its planned path. The YOLOv8 algorithm builds upon the core ideas of YOLOv3 and YOLOv5 and enhances support for image segmentation tasks [15,16]. Through offline model training and online recognition, YOLOv8 can accurately identify and label potholes with their category and confidence score.

In addition to object detection, monocular distance estimation has been widely studied in autonomous driving and mobile robotics. Geometry-based distance decomposition has been used to estimate object distance from factors such as physical height and projected visual height [17]. However, this type of method usually relies on stable object-size priors and is more suitable for raised objects, whereas potholes are low-lying concave targets with irregular boundaries and ambiguous physical height. Ground-plane priors have also been used as effective geometric constraints for monocular depth estimation without requiring extra data sources such as LiDAR, stereo images, or depth maps [18]. Inspired by these studies, this work estimates pothole distance through a ground-plane intersection method. The detected image point is back-projected through the calibrated camera model, and its intersection with the road plane is obtained using road-plane geometric constraints, reducing the dependence on unstable pothole height priors.

The main contributions of this work are as follows: first, a ground-plane intersection method is designed for low-lying pothole targets by combining camera pose information with road-plane geometric constraints, thereby reducing the dependence on unstable pothole height priors; second, sandbox experiments are conducted to evaluate the feasibility, accuracy, and repeatability of the proposed method for short-range pothole distance estimation.

## 2. Monocular Ground Plane Intersection Method

### 2.1. Pothole Detection Algorithm

#### 2.1.1. Algorithm Model and Principle

**Figure 1:**
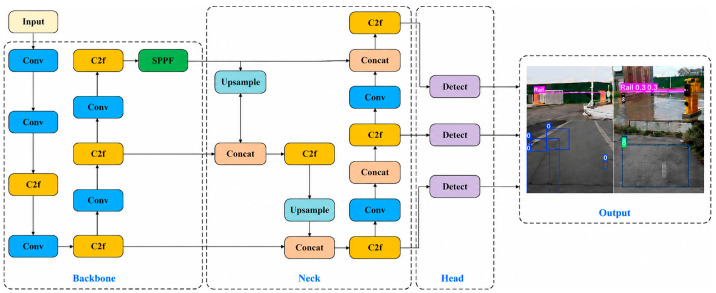
YOLOv8 Network Architecture.

This paper performs pothole detection in two steps. The first step is to detect and identify potholes using an image processing algorithm. The second step is to estimate the distance to these potholes using the ground plane coordinate method. The image recognition algorithm employed in this paper is based on YOLOv8. As shown in Figure 1, the algorithm mainly consists of three parts: a feature extraction network (Backbone), a feature fusion network (Neck), and a detection head (Head). It can perform tasks such as classification, detection, segmentation, and pose estimation. This algorithm uses a new backbone network, a new anchor-free detection head, and a new loss function. It can run on various hardware platforms, from CPUs to GPUs [19].

In the object detection network, the convolutional unit (Conv) extracts local features from the image layer by layer. The Cross-Stage Partial connection and feature fusion unit (C2f) uses grouped convolution and residual connections. This achieves lightweight feature fusion and strengthens gradient flow. The Spatial Pyramid Pooling Fast unit (SPPF) captures multi-scale contextual information through pooling at different scales. The Upsample unit increases the spatial resolution of feature maps using interpolation methods. The Concat operation merges features from different layers along the channel dimension to enable multi-layer feature fusion. Finally, three Detect heads output bounding box coordinates, class probabilities, and object confidence scores on feature maps of different scales. This completes the final object detection [20].

The smart car in this study is controlled by a Raspberry Pi controller. The algorithm is implemented on the Raspberry Pi platform to perform image-based object detection. The system captures environmental images in real time using a camera. It then uses the trained YOLOv8 model to identify and detect potholes within these images and calculates their corresponding distances.

#### 2.1.2. Dataset

A dataset is used for training and evaluating the object detection model to enable automatic recognition and classification of road damage. We employ the GRDDC2020 dataset. GRDDC stands for Global Road Damage Detection Challenge. This dataset is primarily designed for road damage detection [21]. The GRDDC2020 dataset contains a total of 21,041 samples. It covers nine categories of actual road damage conditions. These categories include longitudinal cracks, longitudinal joint seams, transverse cracks, transverse joint seams, alligator cracks, potholes, blurred intersections, blurred white lines, and manhole covers.

**Figure 2:**
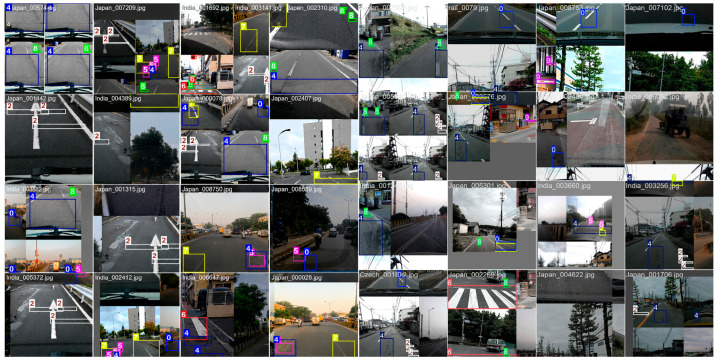
Example of Object Detection Results.

Sample images of the object recognition results are provided. Some images demonstrate the model’s prediction performance on the validation set. In these images, bounding boxes of different colors predict the locations of various pothole categories. Confidence scores are also annotated. This provides a more intuitive view of the model’s training effectiveness. The results are shown in Figure 2.

To further clarify the composition and split of the training data, the dataset was divided into training, validation, and test sets with an approximate ratio of 70:15:15. Since this study mainly involves the recognition of road-surface targets such as potholes and manhole covers, the number and proportion of each road damage category in different dataset splits are summarized in Table 1.

### 2.2. Camera Parameter Calibration

In the field of machine vision, camera calibration is a crucial step. It directly affects the positioning accuracy of the system and the precision of object measurement [22,23]. The camera imaging process involves transformations across four coordinate systems. These four systems are:

(1) World Coordinate System: This represents an object’s coordinates ($X_{w}$, $Y_{w}{,\;Z}_{w}$) in the real three-dimensional space. It is a 3D Cartesian coordinate system used as a reference to describe the spatial positions of both the camera and the target object.
(2) Camera Coordinate System: This is a 3D coordinate system ($X_{c}$ ,$Y_{c}$,$\;Z_{c}$) with the camera’s optical center as its origin. The origin is located at the lens’s optical center. The $X_{c}$ and $Y_{c}$ axes are parallel to the sides of the image plane, respectively. The Z_c axis is the optical axis of the lens, perpendicular to the image plane.
(3) Image Coordinate System: This is a 2D coordinate system (x, y) on the imaging plane where the scene is projected.
(4) Pixel Coordinate System: This is the pixel coordinate system (u, v) of the digital image.

Here, $f_{x}$ and $f_{y}$ represent the focal lengths in pixels along the X and Y axes, respectively. $c_{x}$ and $c_{y}$ are the principal point coordinates, indicating the position of the optical center in the pixel coordinate system. The intrinsic matrix K is a key parameter for monocular distance estimation algorithms. It can be obtained using the Zhang Zhengyou checkerboard calibration method [24]. The Zhang Zhengyou calibration method uses images of a checkerboard calibration pattern. Corresponding image detection algorithms are used to obtain the pixel coordinates (u,v) of each corner point on the pattern. The world coordinate system is fixed on the checkerboard. Consequently, the physical coordinate $Y_{w}$ for any point on the checkerboard is 0. Since the world coordinates of the calibration board are predefined, and the size of each square on the board is known, the physical coordinates $\left(X_{w},Y_{w}=0,Z_{w}\right)$ of each corner point in the world coordinate system can be calculated. The calibration process uses the pixel coordinates (u,v) of each corner point and their corresponding known physical coordinates $\left(X_{w},Y_{w}=0,Z_{w}\right)$ in the world coordinate system. This process determines the camera’s intrinsic and extrinsic matrices, as well as its distortion parameters.

**Figure 3:**
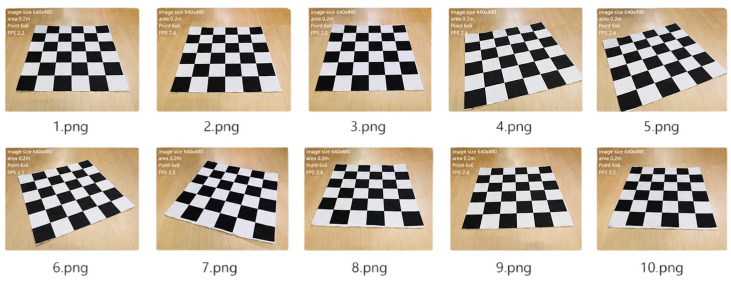
Chessboard calibration images.

We obtained ten images of the checkerboard pattern from different camera poses using the camera capture program. These captured images were then transferred to a PC, where the camera calibration program was executed based on Zhang’s checkerboard calibration method. As shown in Figure 3. The resolution of the calibration images was 640 × 480 pixels. The calibration process provided the camera intrinsic matrix *K*, which serves as a key parameter for the monocular distance estimation algorithm:

(1) $$K=\left[\begin{array}{ccc} 679.38565097 & 0 & 340.90016237\\ 0 & 680.80818677 & 230.54997208\\ 0 & 0 & 1 \end{array}\right]$$

The corresponding distortion coefficients were obtained as:

(2) D=[0.489776,−4.229653,−0.018205,−0.003187,11.744459]

where $D=[k_{1},k_{2},p_{1},p_{2},k_{3}]$, $k_{1}$, $k_{2}$, and $k_{3}$ are the radial distortion coefficients, and $p_{1}$ and $p_{2}$ are the tangential distortion coefficients. The overall RMS reprojection error was 0.466439 pixels, and the mean reprojection error was 0.406487 pixels, with an error range of 0.024623–1.446184 pixels. The mean reprojection errors of the individual calibration images ranged from 0.349794 to 0.462666 pixels, indicating that the calibration results achieved good pixel-level fitting accuracy. In addition, the mean translation vector of the calibration images was $\left[4.244257,-3.247962,\;21.201336\right]$ cm, and the standard deviations along the x, y, and z directions were 3.355368 cm, 3.340989 cm, and 3.190102 cm, respectively. These results demonstrate that the calibration images covered different spatial poses and that the obtained camera parameters provided a reliable geometric basis for subsequent monocular distance estimation.

### 2.3. Pothole Distance Algorithm

This section discusses a new method for estimating distance with a single camera. The method combines geometric projection to achieve pothole distance estimation.

**Figure 4:**
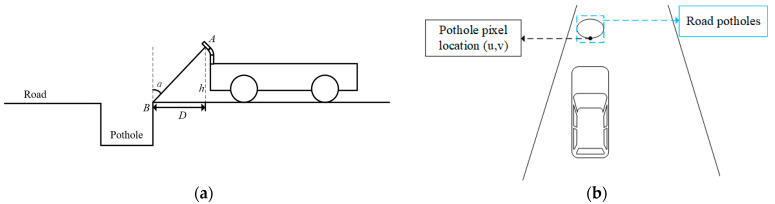
Simplified Model Diagram for Pothole Measurement: (**a**) Side View of Pothole Measurement; (**b**) Top View of Pothole Measurement.

A simplified model for pothole measurement is shown in Figure 4a. Point A is the position of the camera. Point B is the location where the camera first detects the pothole. Distance D is the distance from the vehicle to the pothole at this moment. The letter h represents the installation height of the camera. The angle α is the fixed angle between the camera’s optical axis and the vertical plane. In our system, this angle is set to 30°.

When the camera detects a pothole, it calculates the pothole’s pixel coordinates (u, v), as shown in Figure 4b. To facilitate operations with the camera’s intrinsic matrix, pixel coordinates are usually expressed in homogeneous form as [*u* , *v*, 1]^T^. Here, ‘1’ is a normalized scale factor used to extend the 2D coordinates into 3D homogeneous coordinates. Using the camera’s intrinsic matrix *K* [25,26], we obtain a direction vector ${\vec{d}}_{c}$ in the camera coordinate system. This calculation is shown in Equation (3).

(3) $${\vec{d}}_{c}=K^{-1}\cdot {\left[u,v,1\right]}^{T}$$

At the same time, the camera has a fixed angle α relative to the vertical direction. Therefore, we multiply the direction vector ${\vec{d}}_{c}$ by a rotation matrix. This operation yields a new coordinate system direction, ${\vec{d}}_{w}$, completing the rotation into the world coordinate system. The resulting ${\vec{d}}_{w}$ is a direction vector in the world coordinate system. It points along the ray corresponding to pixel (u, v) and includes components ${[\vec{d}}_{w,x},{\vec{d}}_{w,y},{\vec{d}}_{w,z}]$.

The camera’s position in the world coordinate system is point A = [0, h, 0]. Point B represents the location of the pothole to be detected, with its coordinates denoted as $B=(X_{B},Y_{B},Z_{B})$. The direction from the camera to the pothole can now be represented by the vector $\vec{AB}$.

(4) $$\vec{AB}=A^{T}+\lambda \cdot {\vec{d}}_{w}=A^{T}+\lambda \cdot \left[\begin{array}{ccc} 1 & 0 & 0\\ 0 & \cos(\alpha ) & -\sin(\alpha )\\ 0 & \sin(\alpha ) & \cos(\alpha ) \end{array}\right]\cdot {\vec{d}}_{c}$$

In Equation (4), λ is the unknown parameter to be solved for. In vector $\vec{AB}$, point B is the pothole’s position on the road surface. This gives us the condition $Y_{B}=0$. According to Equation (5):

(5) $$Y_{B}=h+\lambda \cdot {\vec{d}}_{w,y}=0$$

Solving the equation yields

(6) $$\lambda =-\frac{h}{{\vec{d}}_{w,y}}$$

Substituting Equation (6) into Equation (4) allows the components $X_{B}$ and $Z_{B}$ to be obtained, namely:

(7) $$X_{B}=A_{x}+\lambda \cdot {\vec{d}}_{w,x}=-\frac{h\cdot {\vec{d}}_{w,x}}{{\vec{d}}_{w,y}}$$

(8) $$Z_{B}=A_{z}+\lambda \cdot {\vec{d}}_{w,z}=-\frac{h\cdot {\vec{d}}_{w,z}}{{\vec{d}}_{w,y}}$$

Consequently, the coordinates of point P can be obtained.

(9) $$B=(X_{B},Y_{B},Z_{B})=(-\frac{h\cdot {\vec{d}}_{w,x}}{{\vec{d}}_{w,y}},0,-\frac{h\cdot {\vec{d}}_{w,z}}{{\vec{d}}_{w,y}})$$

Finally, the projected length D of segment $\vec{AB}$ is given by:

(10) $$D=\sqrt{{\left(X_{B}-X_{A}\right)}^{2}+{\left(Z_{B}-Z_{A}\right)}^{2}}$$

## 3. Experimental Results and Analysis

This section describes a simulation experiment implemented on a Raspberry Pi. The experiment is based on distance estimation using the ground coordinate system. We use the acquired dataset to evaluate the recognition accuracy for road damage. Experimental validation is also conducted for distance estimation.

### 3.1. Experimental Setup

**Figure 5:**
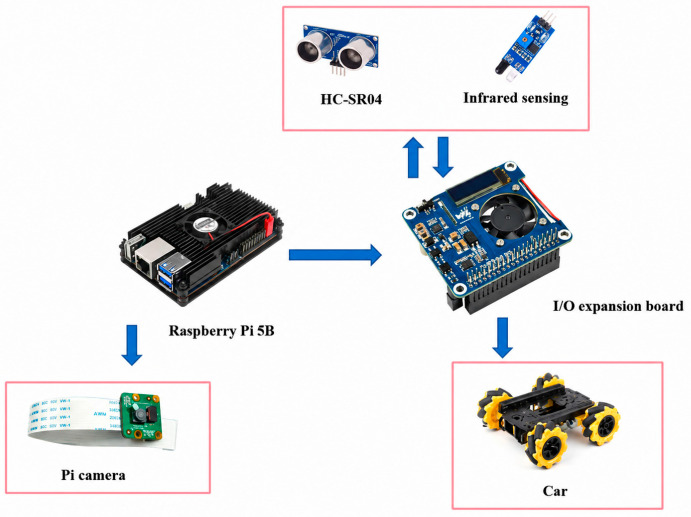
Overall System Design.

The hardware used in this experiment is a smart car system built with a Raspberry Pi 5B. The overall design of the system is shown in Figure 5.

The smart car’s actuation part consists of DC motors, primarily controlling the vehicle’s direction and speed. The Raspberry Pi is equipped with a multi-core processor capable of handling various complex tasks and supports multitasking. Therefore, the CPU used is the Broadcom BCM2712 from the Raspberry Pi 5. This, combined with the Raspberry Pi 5 board and software programming, implements the basic movement functions of the car.

The sensor detection part utilizes a Raspberry Pi 5 board paired with a Raspberry Pi 5-megapixel camera for object detection. Captured images are transmitted to the Raspberry Pi 5 board, where a pothole detection model based on the YOLOv8 object detection algorithm is executed.

**Figure 6:**
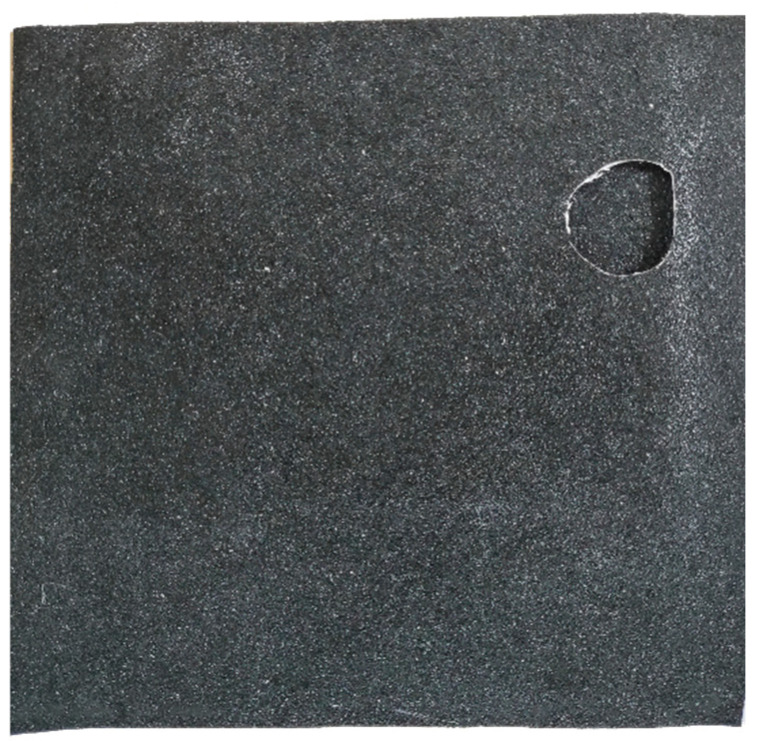
Pothole sandbox model.

The experimental environment was constructed using a sandbox model, as shown in Figure 6.

In this setup, the Raspberry Pi CPU runs a YOLOv8-trained model. Using the camera, it identifies and measures the potholes constructed in the sandbox model, thereby facilitating the collection and testing of experimental data.

### 3.2. Experimental Results

#### 3.2.1. Model Performance

To improve the reproducibility of the model training process, the main configuration and training hyperparameters of the object detection model are summarized in Table 2.

**Figure 7:**
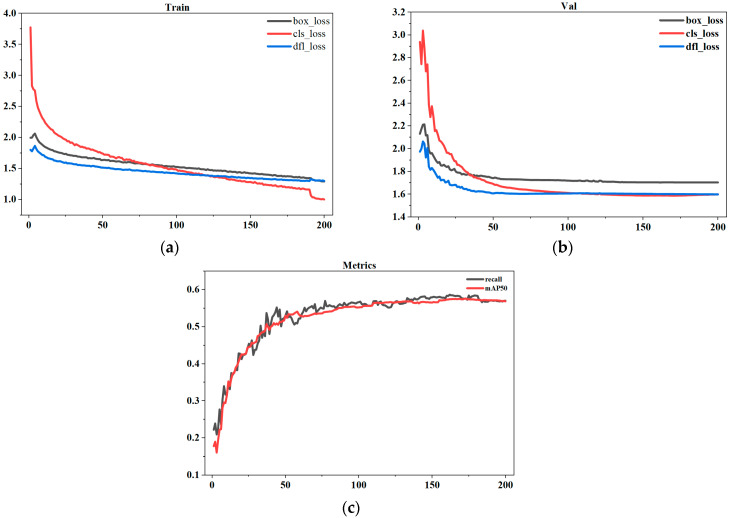
Model performance evaluation curves: (**a**) training curve; (**b**) validation curve; (**c**) metric curves.

The resulting training and validation performance curves are shown in Figure 7.

Figure 7a,b illustrate the evolution of the loss functions during the training and validation processes. The box_loss, cls_loss, and dfl_loss decrease rapidly during the early stages of training and gradually stabilize around 150 epochs. Moreover, the validation loss curves remain highly consistent with those of the training set, indicating excellent convergence and generalization performance, with no evident signs of overfitting. The stepwise decline in classification loss observed in the later stages of training further demonstrates the effectiveness of the optimization strategy in enhancing fine-grained feature extraction and improving classification accuracy.

Figure 7c presents the variation in key evaluation metrics, demonstrating that model performance improves as the number of training epochs increases. Although the metrics exhibit stochastic fluctuations during the upward trend, they gradually stabilize in the later training stage. Based on the final training results, the detection model achieved a recall of 0.5705 and an mAP@50 of 0.5689. These quantitative results indicate that the trained model can provide effective pothole localization for the subsequent short-range distance estimation task. Overall, the model achieves a reasonable balance between detection accuracy and convergence stability under the current training conditions.

#### 3.2.2. Distance Estimation Experiment

**Figure 8:**
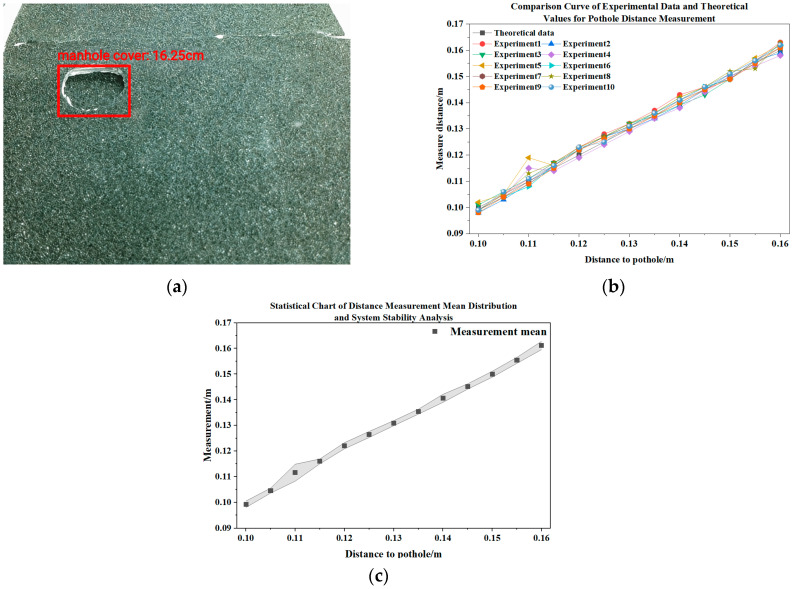
Experimental results of pothole distance estimation: (**a**) pothole detection; (**b**) pothole distance estimation; (**c**) ranging stability.

Before the ranging experiment, the preset distances between the smart-car platform and the simulated pothole were marked using a tape measure at intervals within the range of 0.100–0.160 m. Meanwhile, the camera angle was fixed using a servo mechanism, and the actual camera tilt angle was measured with an angle-measuring instrument to confirm that it reached the preset value of 30°. The smart car was then placed at each marked position to conduct pothole identification and distance estimation experiments. The identification of simulated road potholes within the sandbox model is shown in Figure 8.

The workflow of the proposed system begins with robust target recognition and spatial localization based on a deep learning model. Figure 8a illustrates the inference results of the algorithm in a simulated road environment, demonstrating that the system can accurately capture pothole edge features and provide real-time feedback on target classification and distance estimation, thereby validating the effectiveness of the feature extraction capability under complex textured backgrounds. In determining the effective detection range, physical constraints imposed by the inherent field of view of the monocular camera and near-field imaging characteristics render regions within 0.10 m as a blind zone, where target features cannot be fully extracted to satisfy the requirements for geometric reconstruction. In contrast, a sensing range of up to 0.16 m is sufficient to meet the predefined requirements of this study for short-range obstacle avoidance tasks. Accordingly, a core observation interval of 0.10–0.16 m is established, within which ten independent repeated experiments were conducted to validate the quantitative accuracy of the proposed method.

The ranging accuracy results shown in Figure 8b indicate that the system exhibits an excellent linear mapping relationship over the full measurement range, with experimental measurement curves highly consistent with the theoretical model and only minimal average deviation. Although a slight local deviation is observed around 0.11 m due to minor fluctuations in image feature matching accuracy, the overall consistency of the trend confirms that the algorithm achieves high absolute ranging accuracy. Figure 8c presents the deviation range between the maximum data fluctuation and the mean measurement value obtained from repeated experiments. The deviation remains relatively small and stable throughout the measurement range, providing an intuitive representation of the variation in the measurement results. To further quantitatively evaluate the stability and measurement accuracy of the system, the corresponding statistical results are summarized in Table 3.

Table 3 provides a quantitative evaluation of the measurement performance. Over the theoretical distance range of 0.100–0.160 m, the relative error varies from 0.799% to 3.703%, with an average of 1.8%. Among the 13 measurement points, 11 exhibit relative errors below 3%, accounting for 84.6% of all measurements. The standard deviation ranges from 1.4 to 5.5 mm, with an average of approximately 2.18 mm. Except for the result at 0.110 m, the standard deviations at all other measurement points do not exceed 2.6 mm. Therefore, the low average relative error and the concentrated standard deviation values quantitatively demonstrate that the system can maintain stable measurement performance over the selected detection range.

From Table 4, it can be seen that the system operates stably overall during the processes of pothole identification and distance calculation. After processing a large amount of pothole data, the camera’s average time consumption for identifying a pothole is 0.552 s. The average time consumption for calculating the distance after identification is 0.016 s. This indicates that the distance calculation module has a high operational speed. Overall, the main time cost of the system is concentrated in the image recognition stage, while the distance calculation stage accounts for a smaller proportion of the overall time consumption.

It should be noted that the main focus of this study is the pothole distance-estimation algorithm after pothole localization, rather than the lightweight optimization of the front-end object detection network. In the current implementation, the lightweight YOLOv8n model was adopted to reduce the computational load of the image recognition stage, while the proposed geometric distance-estimation module only requires simple coordinate transformation and ground-plane intersection calculation. Therefore, the additional computational overhead introduced by the distance-estimation module is relatively small. For future dynamic vehicle-mounted applications, the real-time performance can be further improved by optimizing the front-end detection module, such as model quantization, model pruning, input-resolution adjustment, hardware acceleration, and asynchronous processing. These optimization strategies will be considered in future work.

In summary, the experimental results verify the feasibility of the monocular vision-based pothole ranging method for short-range distance estimation under the controlled sandbox conditions of this study. However, the current validation is still limited to a controlled sandbox environment, and further optimization and validation under dynamic vehicle-mounted scenarios are needed in future work.

### 3.3. Performance Comparison

To verify the accuracy of the proposed method, an ultrasonic module was also used concurrently in the experiments for comparison. Ultrasonic testing is a commonly used non-destructive testing technology. It acquires target information through the propagation characteristics of sound waves in a medium and can complete measurements relatively quickly.

The basic principle of ultrasonic distance measurement follows a “transmit-reflect-receive” operating mode. The specific process is as follows: first, the ultrasonic module emits a signal of a specific frequency towards the target direction through its transmitter. When this ultrasonic wave encounters a pothole, which is oriented parallel to the transmitter, it reflects. The reflected wave then travels back in the opposite direction. Subsequently, the module’s receiver captures the returning ultrasonic signal. By measuring the time difference between emission and reception, the system calculates the actual distance between the pothole and the sensor.

**Figure 9:**
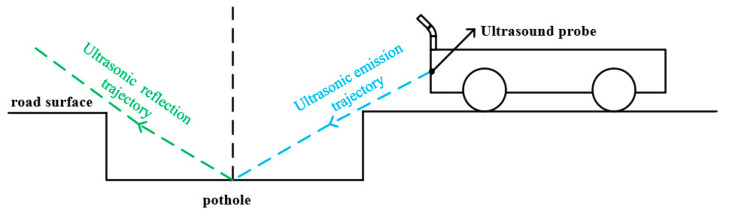
Model of Ultrasonic Pothole Measurement.

In Figure 9, due to the specific nature of a pothole, when the ultrasonic wave encounters it, the reflection direction deviates away from the ultrasonic receiver. The reflected wave cannot be captured by the receiver. In this situation, because the ultrasonic receiver fails to detect any reflected signal, the system cannot calculate an accurate time difference. Consequently, it cannot correctly compute the distance between the pothole and the sensor, leading to a ranging failure. Therefore, ultrasonic technology cannot effectively accomplish the task of pothole distance measurement.

Table 5 summarizes the comparative results between the proposed method and existing pothole ranging methods. It should be noted that the deviation rates of the VIDAR method and the binocular vision method were obtained from relevant studies. Since different studies vary in sensor configuration, experimental scenarios, ranging intervals, and data acquisition conditions, the results in Table 5 are mainly used as a literature-based reference comparison of different ranging methods, rather than as a strict benchmark test under identical conditions. As shown in Table 5, the proposed ground-plane coordinate system method achieves a relatively low ranging deviation rate in comparison with existing studies, indicating that the proposed method has certain advantages in pothole distance estimation accuracy.

**Figure 10:**
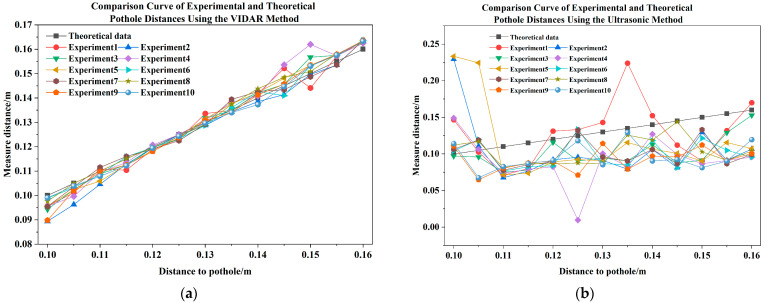
Comparison between measured and theoretical pothole distances under identical experimental conditions: (**a**) VIDAR method; (**b**) ultrasonic ranging method.

To further improve the comparability of the experimental results, additional comparative experiments using the ultrasonic ranging method and the VIDAR method were conducted under the same experimental environment as the proposed method. These experiments adopted consistent testing conditions, including the same pothole scenario, measurement distance range, and experimental setup, so as to further evaluate the ranging accuracy and stability of the proposed method under unified testing conditions. The ranging results are shown in Figure 10 and Table 6. Figure 10 presents the comparison between the measured results obtained by the two ranging methods and the theoretical values, while Table 6 further summarizes the corresponding mean measured distance, relative error, and standard deviation.

Figure 10 further presents the ranging results of the two comparison methods under the same experimental conditions as those used for the proposed method in Figure 8. As shown in Figure 10a, the VIDAR method generally follows the increasing trend of the theoretical distance, although certain deviations are observed at several measurement points. In contrast, the ultrasonic ranging method shown in Figure 10b exhibits larger fluctuations, indicating relatively weaker stability in short-range pothole distance estimation. To further quantify the ranging performance of these two comparison methods, the corresponding statistical results are summarized in Table 6.

As shown in Table 5, the proposed ground-plane coordinate system method achieves measurement results that are generally close to the theoretical distances within the range of 0.100–0.160 m. The relative error remains at a low level for most measurement points, with an average relative error of approximately 1.8%. Meanwhile, the average standard deviation is about 2.185 mm, indicating that the proposed method has good repeatability and stability in short-range pothole distance estimation.

For comparison, VIDAR and ultrasonic ranging methods were further tested under the same experimental conditions. The VIDAR method also shows a relatively low average relative error, but its measurement results exhibit larger deviations at several distance points compared with the proposed method. In contrast, the ultrasonic ranging method shows significantly larger errors and stronger fluctuations, with its measured values deviating markedly from the theoretical distances. This indicates that ultrasonic ranging is more susceptible to the pothole surface morphology, reflection angle, and sensor response characteristics in short-range pothole measurement scenarios.

**Figure 11:**
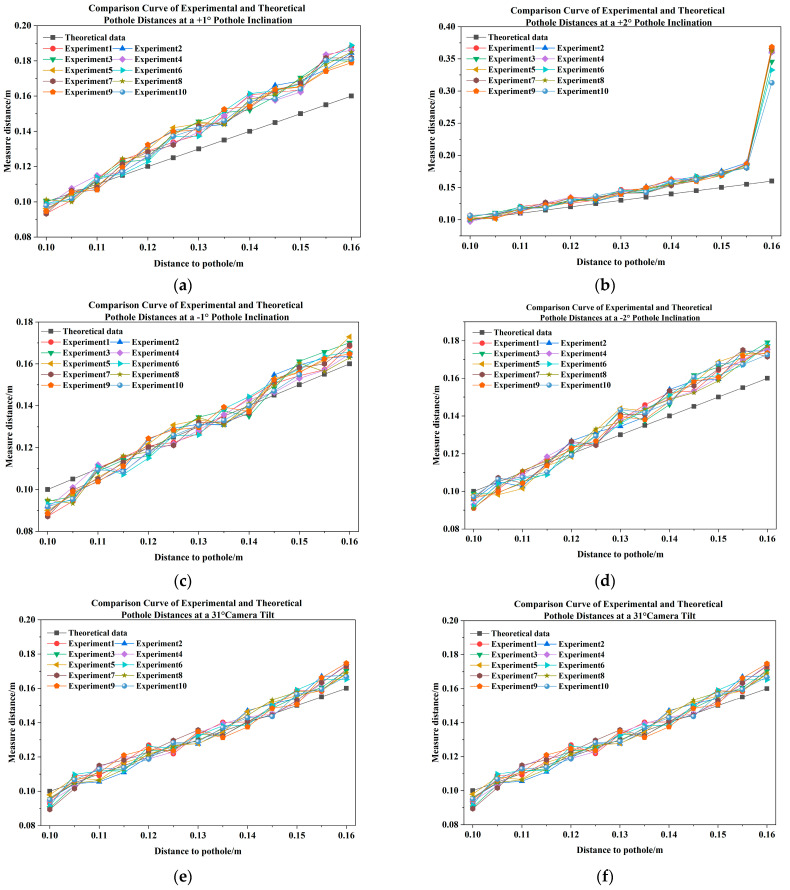
Robustness analysis of pothole distance estimation under road-surface inclination and camera-tilt perturbations: (**a**) comparison between measured and theoretical distances at a +1° road-surface inclination; (**b**) comparison between measured and theoretical distances at a +2° road-surface inclination; (**c**) comparison between measured and theoretical distances at a −1° road-surface inclination; (**d**) comparison between measured and theoretical distances at a −2° road-surface inclination; (**e**) comparison between measured and theoretical distances when the camera tilt angle increases from 30° to 31°; (**f**) comparison between measured and theoretical distances when the camera tilt angle decreases from 30° to 29°.

Overall, compared with the two comparative methods, the proposed ground-plane coordinate system method achieves a better balance between measurement accuracy and stability, indicating that it is more suitable for pothole distance estimation under the experimental conditions of this study. To further verify the robustness and intrinsic stability of the proposed method, additional perturbation experiments were conducted under different road-surface inclination and camera-tilt conditions, and the corresponding results are presented in Figure 11 and Table 7, Table 8, Table 9, Table 10, Table 11 and Table 12.

Figure 11 presents the robustness analysis of the proposed pothole distance estimation method under road-surface inclination and camera-tilt perturbations. Figure 11a–d show the comparison between the measured and theoretical pothole distances when the road-surface inclination is set to +1°, +2°, −1°, and −2°, respectively. In general, the measured curves maintain a consistent increasing trend with the theoretical distance, indicating that the proposed geometric model can still provide stable distance estimation under moderate road-slope variations. Compared with the ±1° cases, the ±2° inclination conditions introduce relatively larger deviations, especially at longer measurement distances, suggesting that the ranging error increases as the road-surface inclination becomes more pronounced. Figure 11e,f further show the ranging results when the camera tilt angle deviates from the nominal 30° installation angle by +1° and −1°, respectively. The experimental curves remain close to the theoretical curve, demonstrating that the proposed method has a certain tolerance to small camera-angle drift. Overall, the results in Figure 11 indicate that the ground-plane intersection method maintains acceptable robustness under road-surface inclination variations within ±2° and camera-tilt perturbations within ±1°. To provide a more detailed quantitative evaluation of the measurement accuracy and stability under these perturbation conditions, the corresponding statistical results are summarized in Table 7, Table 8, Table 9, Table 10, Table 11 and Table 12.

Figure 11 and Table 7, Table 8, Table 9, Table 10, Table 11 and Table 12 further evaluate the robustness of the proposed method under road-surface inclination and camera-tilt perturbations. Under road-surface inclination conditions, the measured distances generally maintain the same increasing trend as the theoretical distances, but the relative error increases when the inclination becomes more pronounced. The average relative errors under +1°, +2°, −1°, and −2° road-surface inclinations are 8.38%, 9.16%, 3.16%, and 5.63%, respectively, indicating that positive road-surface inclination has a stronger influence on the ranging accuracy than negative inclination in the current experimental setup. For camera-tilt perturbations, the average relative errors at 31° and 29° are 2.34% and 5.03%, respectively, showing that the proposed model is less sensitive to a ±1° camera-angle drift than to road-surface inclination changes. Meanwhile, the standard deviations under all perturbation conditions remain stable at approximately 3 mm, with the maximum value not exceeding 3.6 mm. These results demonstrate that although slope and camera-angle perturbations introduce certain systematic deviations, the proposed method still maintains good repeatability and acceptable robustness within the tested perturbation range.

In summary, the experimental results show that ultrasonic ranging is strongly affected by reflection angle, pothole morphology, and ground interference, resulting in large fluctuations and obvious deviations from the true distance. In contrast, camera-based visual ranging methods can provide more stable measurements for short-range pothole distance estimation. Compared with the VIDAR and ultrasonic methods, the proposed ground-plane coordinate system method achieves a better balance between measurement accuracy, stability, and computational efficiency. Furthermore, the perturbation experiments under different road-surface inclination and camera-tilt conditions demonstrate that the proposed method maintains good repeatability and acceptable robustness within the tested range. It should be noted that the proposed geometric model is based on a calibrated camera with a fixed installation height and a fixed nominal tilt angle of 30°, and the road surface near the pothole is approximated as a local ground plane. Under these experimental assumptions and controlled sandbox conditions, the proposed method can achieve stable short-range pothole distance estimation. Therefore, the proposed method is suitable for short-range pothole distance estimation under the experimental conditions of this study, while compensation for larger road-slope variations, camera-pose changes, and more complex real-road conditions should be further considered in future practical applications.

## 4. Conclusions

This study proposes a YOLOv8-based monocular ground-plane intersection method for short-range pothole detection and distance estimation. By combining pothole recognition with calibrated camera geometry, the detected pothole position in the image is projected onto the ground plane to estimate its distance from the smart-car platform. The method was implemented on a Raspberry Pi 5B-based low-cost experimental platform and evaluated in a controlled sandbox environment. The experimental results show that the proposed method can achieve effective pothole detection and distance estimation within the tested short-range interval of 0.10–0.16 m. Compared with ultrasonic ranging and the VIDAR method under the same experimental conditions, the proposed ground-plane coordinate system method provides more stable measurement results and a lower average relative error.

Additional perturbation experiments further demonstrate that the method maintains good repeatability under road-surface inclination variations within ±2° and camera-tilt perturbations within ±1°, although larger slope changes may introduce systematic ranging deviations. Overall, this work verifies the preliminary feasibility of using a monocular vision-based ground-plane intersection model for short-range pothole distance estimation in a controlled experimental setting. The results indicate that the proposed method has advantages in terms of low hardware cost, simple geometric modeling, and stable repeated measurements. However, the current validation is still limited to a sandbox-based experimental environment. Future work will focus on expanding the range of controlled test conditions, improving error compensation for road-surface inclination and camera-pose variation, and enhancing the robustness of the detection and ranging framework under more diverse experimental scenarios.

## Figures and Tables

**Table 1:** Category Distribution of the GRDDC2020 Dataset Used for Model Training.

Class	Meaning	Train	Val	Test	Total	Proportion (%)
d00	Longitudinal crack	4657	956	979	6592	19.00
d01	Longitudinal construction joint	107	41	41	179	0.52
d10	Transverse crack	3097	659	690	4446	12.81
d11	Transverse construction joint	31	7	7	45	0.13
d20	Alligator crack	5827	1336	1218	8381	24.15
d40	Pothole	3944	850	833	5627	16.22
d43	Blurred crosswalk	575	109	109	793	2.29
d44	Blurred white line	3519	760	778	5057	14.57
d50	Manhole cover	2549	501	531	3581	10.32
Total	All categories	24,306	5219	5176	34,701	100.00

**Table 2:** Main configuration and training hyperparameters of the YOLOv8 model.

Parameter	Setting
Model variant	YOLOv8n
Input image size	640 × 640 pixels
Training epochs	200
Batch size	16
Optimizer	SGD
Initial learning rate	0.01
Weight decay	0.0001

**Table 3:** Statistical results of distance measurement accuracy and stability.

Theoretical Distance/m	Mean Measured Distance/m	Relative Error/%	SD/mm
0.100	0.099	2.200	2.1
0.105	0.104	1.329	1.8
0.110	0.113	3.627	5.5
0.115	0.117	1.639	1.5
0.120	0.123	3.703	2.2
0.125	0.127	1.820	1.7
0.130	0.132	1.362	1.6
0.135	0.136	0.799	1.4
0.140	0.141	1.543	2.5
0.145	0.146	1.034	1.8
0.150	0.150	1.067	2.1
0.155	0.156	1.032	1.6
0.160	0.162	1.675	2.6

**Table 4:** Time Consumption for Pothole Detection and Distance Estimation.

Obstacle Category	Average Time for Pothole Detection	Average Time for Distance Estimation After Detection
Pothole	0.552 s	0.016 s

**Table 5:** Literature-based comparison of pothole distance measurement methods.

Method	Deviation Rate
VIDAR method [27]	4.75%
Binocular vision method [28]	2.04%
Ground-plane coordinate system method (our method)	1.8%

**Table 6:** Comparison of pothole distance measurements.

Ranging Method	Theoretical Distance/m	Mean Measured Distance/m	Relative Error/%	SD/mm
VIDAR method	0.100	0.095	4.880	3.3
	0.105	0.102	2.886	2.5
	0.110	0.109	1.573	2.1
	0.115	0.113	1.774	1.8
	0.120	0.119	0.692	0.7
	0.125	0.124	0.944	0.9
	0.130	0.130	0.977	1.6
	0.135	0.136	1.237	2.0
	0.140	0.141	1.521	2.0
	0.145	0.146	2.290	4.2
	0.150	0.153	2.687	4.8
	0.155	0.157	1.529	1.7
	0.160	0.163	2.012	0.5
Ultrasonic method	0.100	0.140	40.952	51.0
	0.105	0.113	24.401	43.9
	0.110	0.076	31.062	4.9
	0.115	0.082	28.931	4.9
	0.120	0.095	22.506	15.7
	0.125	0.096	26.718	37.7
	0.130	0.099	25.965	17.7
	0.135	0.110	31.552	44.3
	0.140	0.114	20.613	17.3
	0.145	0.098	32.288	18.7
	0.150	0.104	30.687	18.9
	0.155	0.102	33.948	17.2
	0.160	0.115	29.223	25.6

**Table 7:** Statistical results of pothole distance estimation at a +1° road-surface inclination.

Theoretical Distance/m	Mean Measured Distance/m	Relative Error/%	SD/mm
0.1	0.097	3.11	2.9
0.105	0.104	1.362	2.5
0.11	0.111	0.845	3
0.115	0.12	4.409	3.1
0.12	0.128	6.283	3.2
0.125	0.137	9.8	3.3
0.13	0.142	9.077	2.9
0.135	0.148	9.348	3.3
0.14	0.157	11.993	3.2
0.145	0.162	11.669	2.7
0.15	0.166	10.92	2.7
0.155	0.179	15.49	3.4
0.16	0.183	14.656	3.6

**Table 8:** Statistical results of pothole distance estimation at a +2° road-surface inclination.

Theoretical Distance/m	Mean Measured Distance/m	Relative Error/%	SD/mm
0.1	0.102	1.89	3.2
0.105	0.106	1.057	3.3
0.11	0.117	6	2.9
0.115	0.122	6.07	3.3
0.12	0.13	8.5	3.2
0.125	0.133	6.096	2.7
0.13	0.142	9.608	2.7
0.135	0.146	8.496	3.4
0.14	0.158	12.643	3.6
0.145	0.163	12.6	2.9
0.15	0.171	14.28	2.5
0.155	0.184	18.987	3
0.16	0.365	12.788	3.1

**Table 9:** Statistical results of pothole distance estimation at a −1° road-surface inclination.

Theoretical Distance/m	Mean Measured Distance/m	Relative Error/%	SD/mm
0.1	0.091	9.23	2.9
0.105	0.097	7.629	2.5
0.11	0.108	1.955	3
0.115	0.112	2.904	3.1
0.12	0.12	0.317	3.2
0.125	0.126	0.792	3.3
0.13	0.131	0.523	2.9
0.135	0.134	0.43	3.3
0.14	0.14	0.214	3.2
0.145	0.151	3.821	2.7
0.15	0.157	4.713	2.7
0.155	0.161	3.955	3.4
0.16	0.167	4.65	3.6

**Table 10:** Statistical results of pothole distance estimation at a −2° road-surface inclination.

Theoretical Distance/m	Mean Measured Distance/m	Relative Error/%	SD/mm
0.1	0.095	5.11	2.9
0.105	0.103	2.229	3.1
0.11	0.106	3.464	3.2
0.115	0.114	1.139	3.3
0.12	0.123	2.517	2.9
0.125	0.128	2.712	3.3
0.13	0.139	7.215	3.2
0.135	0.142	4.904	2.7
0.14	0.15	7.157	2.7
0.145	0.157	8.366	3.4
0.15	0.163	8.827	3.6
0.155	0.171	10.174	2.9
0.16	0.175	9.431	2.5

**Table 11:** Statistical results of pothole distance estimation at a 31° camera tilt angle.

Theoretical Distance/m	Mean Measured Distance/m	Relative Error/%	SD/mm
0.1	0.094	6.28	2.7
0.105	0.106	0.638	2.7
0.11	0.11	0.355	3.4
0.115	0.116	0.47	3.6
0.12	0.122	2.075	2.9
0.125	0.126	0.472	2.5
0.13	0.132	1.315	2.9
0.135	0.136	0.504	3.1
0.14	0.142	1.536	3.2
0.145	0.148	2.407	3.3
0.15	0.155	3.5	2.9
0.155	0.162	4.6	3.3
0.16	0.17	6.269	3.2

**Table 12:** Statistical results of pothole distance estimation at a 29° camera tilt angle.

Theoretical Distance/m	Mean Measured Distance/m	Relative Error/%	SD/mm
0.1	0.079	20.9	2.9
0.105	0.092	12.448	3.3
0.11	0.102	7.464	3.2
0.115	0.108	5.983	2.7
0.12	0.112	6.442	2.7
0.125	0.123	1.752	3.4
0.13	0.128	1.638	3.6
0.135	0.13	3.504	2.9
0.14	0.14	0.193	2.5
0.145	0.146	0.89	2.9
0.15	0.152	1.36	3.1
0.155	0.159	2.419	3.2
0.16	0.161	0.45	3.3

## Data Availability

The data used in this study are openly available in the Mendeley Data repository under the dataset “RDD2020: An Image Dataset for Smartphone-based Road Damage Detection and Classification” at https://data.mendeley.com/datasets/5ty2wb6gvg/1 (accessed on 20 June 2026; DOI: 10.17632/5ty2wb6gvg.1).
